# Supplementary material for: Lichen mimesis in mid-Mesozoic lacewings
Source: eLife. 2020 Jul 29;9:e59007. doi: 10.7554/eLife.59007 (PMC7462608; doi:10.7554/eLife.59007)
Supplement: Supplementary file 1. [file elife-59007-supp1.docx]

**Supplementary file 1**

| **Table S1: Branch width of forewing pattern of *Lichenipolystoechotes ramimaculatus* and lichen thallus of *Daohugouthallus ciliiferus*** | | | |
| --- | --- | --- | --- |
| **Number** | **Forewing pattern branch width**  **of *Lichenipolystoechotes ramimaculatus***  **(CNU-NEU-NN2019004P) (Figure 4**–**figure supplement 1A)** | **Thallus width of**  ***Daohugouthallus ciliiferus***  **(PB23120) (Figure 4**–**figure supplement 1B)** | **Thallus width of**  ***Daohugouthallus ciliiferus***  **(B0474) (Figure 4**–**figure supplement 1C)** |
| 1 | 0.718 | 0.995 | 1.372 |
| 2 | 0.837 | 0.556 | 1.246 |
| 3 | 0.671 | 0.859 | 0.963 |
| 4 | 1.297 | 0.933 | 0.97 |
| 5 | 0.943 | 1.471 | 0.795 |
| 6 | 0.626 | 0.938 | 0.957 |
| 7 | 1.114 | 1.211 | 0.862 |
| 8 | 1.227 | 1.13 | 0.976 |
| 9 | 1.149 | 1.034 | 0.911 |
| 10 | 0.75 | 1.234 | 1.236 |
| 11 | 1.071 | 0.983 | 1.211 |
| 12 | 1.085 | 0.966 | 1.222 |
| 13 | 1.238 | 0.879 | 1.007 |
| 14 | 1.099 | 0.636 | 1.367 |
| 15 | 0.841 | 0.811 | 1.154 |
| 16 | 1.094 | 1.244 | 1.208 |
| 17 | 0.815 | 0.728 | 1.155 |
| 18 | 1.096 | 0.625 | 0.972 |
| 19 | 1.107 | 0.812 | 1.064 |
| 20 | 1.484 | 0.557 | 1.286 |
| 21 | 1.354 | 0.691 | 0.738 |
| 22 | 1.381 | 1.189 | 0.816 |
| 23 | 0.786 | 1.298 | 0.614 |
| 24 | 0.667 | 1.722 | 0.97 |
| 25 | 1.354 | 1.536 | 0.644 |
| 26 | 0.817 | 0.698 | 0.858 |
| 27 | 1.763 | 0.474 | 0.849 |
| 28 | 1.049 | 0.59 | 0.809 |
| 29 | 1.017 | 0.734 | 0.697 |
| 30 | 0.764 | 1.051 | 0.557 |
| 31 |  | 0.967 | 0.94 |
| 32 |  | 1.196 | 1.035 |
| 33 |  | 1.614 | 0.639 |
| 34 |  | 1.349 | 0.857 |
| 35 |  | 1.065 | 0.939 |
| 36 |  | 0.503 | 1.388 |
| 37 |  | 0.894 | 1.244 |
| 38 |  | 0.666 | 0.95 |
| 39 |  | 0.858 | 0.705 |
| 40 |  | 0.871 | 0.921 |
| 41 |  | 0.938 | 1.019 |
| 42 |  | 0.66 | 0.627 |
| 43 |  | 0.823 | 0.857 |
| 44 |  | 0.593 | 1.03 |
| 45 |  | 0.852 | 1.202 |
| 46 |  | 0.732 | 1.18 |
| 47 |  | 0.545 | 0.919 |
| 48 |  | 0.778 | 0.856 |
| 49 |  | 0.79 | 1.279 |
| 50 |  | 0.855 | 1.302 |
| 51 |  | 0.855 | 0.967 |
| 52 |  | 1.495 | 0.914 |
| 53 |  | 0.899 | 1.166 |
| 54 |  | 1.353 | 1.215 |
| 55 |  | 1.199 | 1.464 |
| 56 |  | 1.404 | 1.116 |
| 57 |  | 1.219 | 0.967 |
| 58 |  | 1.272 | 0.832 |
| 59 |  | 1.195 | 0.951 |
| 60 |  | 0.903 | 1.348 |
| 61 |  | 1.166 | 1.126 |
| 62 |  | 1.201 | 1.585 |
| 63 |  | 0.954 | 1.455 |
| 64 |  | 1.391 | 1.361 |
| 65 |  | 1.049 | 0.882 |
| 66 |  | 1.27 | 0.684 |
| 67 |  | 1.758 | 0.804 |
| 68 |  | 1.619 | 0.749 |
| 69 |  | 1.256 | 0.813 |
| 70 |  | 1.387 | 0.89 |
| 71 |  |  | 0.717 |
| 72 |  |  | 1.199 |
| 73 |  |  | 0.844 |
| 74 |  |  | 1.019 |
| 75 |  |  | 0.976 |
| 76 |  |  | 0.895 |
| 77 |  |  | 0.723 |
| 78 |  |  | 0.949 |
| 79 |  |  | 0.977 |
| 80 |  |  | 0.953 |
| **Range** | 0.626–1.763 | 0.474–1.758 | 0.557–1.585 |
| **Average** | 1.040466667 | 1.013985714 | 0.99895 |
|  |  |  |  |
| **Notes:** | | | |
| Measurements are in millimeters. | | | |
| The measuring lines are shown in the Figure 4–figure supplement 1 and the results are arranged randomly. | | | |
|  |  |  |  |
