# Supplementary material for: Lichen mimesis in mid-Mesozoic lacewings
Source: eLife. 2020 Jul 29;9:e59007. doi: 10.7554/eLife.59007 (PMC7462608; doi:10.7554/eLife.59007)
Supplement: Transparent reporting form [file elife-59007-transrepform.pdf]

eLIFE
1st Floor
24 Hills Road
Cambridge CB2 1JP, UK
P 01223 855340
W elifesciences.org
T @elife

eLife's transparent reporting form

We encourage authors to provide detailed information *within their submission* to facilitate the interpretation and replication of experiments. Authors can upload supporting documentation to indicate the use of appropriate reporting guidelines for health-related research (see EQUATOR Network), life science research (see the BioSharing Information Resource), or the ARRIVE guidelines for reporting work involving animal research. Where applicable, authors should refer to any relevant reporting standards documents in this form.

If you have any questions, please consult our Journal Policies and/or contact us: editorial@elifesciences.org.

Sample-size estimation

- You should state whether an appropriate sample size was computed when the study was being designed
- You should state the statistical method of sample size computation and any required assumptions
- If no explicit power analysis was used, you should describe how you decided what sample (replicate) size (number) to use

Please outline where this information can be found within the submission (e.g., sections or figure legends), or explain why this information doesn't apply to your submission:

All the samples in this study are compression fossils from near Daohugou Village, Inner Mongolia Autonomous Region, China. The number of samples is idiosyncratic and is dictated by the vagaries of the fossil record, which often offers single or a few specimens of the same species. In terms of measurements of the sample, the size of the specimens examined were sufficient for light microscopy (larger sized samples), which were examined and photographed using a Nikon SMZ25 microscope attached to a Nikon DS-Ri2 digital camera system. Detailed information can be seen in the Materials and methods section and figures in the main text.

Replicates

- You should report how often each experiment was performed
- You should include a definition of biological versus technical replication
- The data obtained should be provided and sufficient information should be provided to indicate the number of independent biological and/or technical replicates
- If you encountered any outliers, you should describe how these were handled
- Criteria for exclusion/inclusion of data should be clearly stated
- High-throughput sequence data should be uploaded before submission, with a private link for reviewers provided (these are available from both GEO and ArrayExpress)

Please outline where this information can be found within the submission (e.g., sections or figure legends), or explain why this information doesn't apply to your submission:

eLife Sciences Publications, Ltd is a limited liability non-profit non-stock corporation incorporated in the State of Delaware, USA, with company number 5030732, and is registered in the UK with company number FC030576 and branch number BR015634 at the address 1st Floor, 24 Hills Road, Cambridge CB2 1JP | August 2014

In this study, we used microtomy and scanning electron microscopy to detect the hypha and chlorella cells of the lichen fossil. For the verification that this association is indeed mimicry, we used box plots of measurements to show the lower and upper extremes and averages of branch widths of insect's forewing pattern and the corresponding lichen thallus. Detailed information can be seen in the Materials and methods section and figure legends in the main text and Supplementary file 1.

Statistical reporting

- Statistical analysis methods should be described and justified
- Raw data should be presented in figures whenever informative to do so (typically when N per group is less than 10)
- For each experiment, you should identify the statistical tests used, exact values of N, definitions of center, methods of multiple test correction, and dispersion and precision measures (e.g., mean, median, SD, SEM, confidence intervals; and, for the major substantive results, a measure of effect size (e.g., Pearson's r, Cohen's d)
- Report exact p-values wherever possible alongside the summary statistics and 95% confidence intervals. These should be reported for all key questions and not only when the p-value is less than 0.05.

Please outline where this information can be found within the submission (e.g., sections or figure legends), or explain why this information doesn't apply to your submission:

Statistical method for mimicry verification is used in this research. We used box plots in Origin 2018 software to relate the extremes and averages of branch widths of insect's forewing pattern to the lichen thallus. Detailed information can be seen in the Materials and methods section and Figure 4 and Figure 4-figure supplement 1 in the main text and Supplementary file 1.

Group allocation

- Indicate how samples were allocated into experimental groups (in the case of clinical studies, please specify allocation to treatment method); if randomization was used, please also state if restricted randomization was applied
- Indicate if masking was used during group allocation, data collection and/or data analysis

Please outline where this information can be found within the submission (e.g., sections or figure legends), or explain why this information doesn't apply to your submission:

There is no group allocation in this study.

Additional data files ("source data")

- We encourage you to upload relevant additional data files, such as numerical data that are represented as a graph in a figure, or as a summary table
- Where provided, these should be in the most useful format, and they can be uploaded as "Source data" files linked to a main figure or table
- Include model definition files including the full list of parameters used
- Include code used for data analysis (e.g., R, MatLab)
- Avoid stating that data files are "available upon request"

Please indicate the figures or tables for which source data files have been provided:

Supplementary file 1 is a table representing all the measured values plotted in Figure 4F and Figure 4-figure Supplement 1.
